# First-in-Class Immuno-Oncology Drug APG157DS Repolarizes Innate Immune Cells and Induces Durable Remission in a Syngeneic Glioblastoma Model

**DOI:** 10.3390/ijms27156687

**Published:** 2026-07-27

**Authors:** Shubhasmita Mohapatra, Adrian Guerrero, Neha Rahman, Stefan Markovic, Lauren O’Donnell, Youssef Zaim Wadghiri, Khondoker Takia Zaman, Luis Avila, Parag Mehta, Probal Banerjee

**Affiliations:** 1 Department of Chemistry, The College of Staten Island (CUNY), Staten Island, NY 10314, USA; shubhasmitamohapatra@gmail.com; 2 Doctoral Program in Biochemistry, The College of Staten Island (CUNY), New York, NY 10314, USA; adrian.guerrero.cordova@gmail.com (A.G.); takiazaman@gmail.com (K.T.Z.); 3 Department of Radiology, Bernard and Irene Schwartz Center for Biomedical Imaging & Center for Advanced Imaging Innovation and Research and Preclinical Imaging, Division for Advanced Research Technologies, New York University Grossman School of Medicine, New York, NY 10016, USA; neha.rahman@nyulangone.org (N.R.); stefan.markovic@nyulangone.org (S.M.); youssef.zaimwadghiri@nyulangone.org (Y.Z.W.); 4 Aveta Biomics, Inc., 110 Great Road, Suite 302, Bedford, MA 01730, USA; lavila@avetabiomics.com (L.A.); pmehta@avetabiomics.com (P.M.); 5 Center for Developmental Neuroscience, The College of Staten Island (CUNY), Staten Island, NY 10314, USA

**Keywords:** APG157DS, curcumin, glioblastoma, tumor-associated microglia and macrophages (TAMs), M2/M1, Arg1/iNOS

## Abstract

APG157DS is a multi-component investigational drug which is currently the subject of multiple cancer trials. It has shown promising efficacy in patients with head and neck cancer. We report its immuno-modulatory effect in a syngeneic mouse model of glioblastoma (GBM). Long-term treatment with APG157DS led to durable tumor remission in 50% of mice, while all vehicle-treated mice reached humane endpoints within 42 days. Mechanistically, the drug induced selective repolarization of tumor-associated macrophages (TAMs) from an immunosuppressive Arg1^high^/iNOS^low^ phenotype to a tumoricidal Arg1^low^/iNOS^high^ state, accompanied by increased intratumoral recruitment of activated (NKp46+) natural killer cells and CD8+ cytotoxic T-cells. APG157DS also suppressed vascular endothelial growth factor (VEGF) and Hypoxia-inducible factor 1-alpha (HIF-1α) expression in tumors, further impairing tumor growth. Notably, APG157DS did not elicit off-target macrophage activation in peripheral tissues such as the spleen, highlighting its selective immune targeting. These findings provide a mechanistic rationale for further clinical development of APG157DS in GBM and potentially other immune-evasive cancers.

## 1. Introduction

APG157DS is a novel, orally administered immuno-oncology drug [1]. It is being investigated as a single agent in head and neck cancer [2,3] and in combination with anti-VEGF drug bevacizumab for recurrent glioma. The drug contains a key zinflavonoid (1E,6E)-1,7-bis(4-hydroxy-3-methoxyphenyl)-1,6-heptadiene-3,5-dione, also known as diferuloylmethane (DFM). The anticancer activity of DFM has been reported in many publications, but the earlier generations of drugs with DFM did not yield convincing results in clinical trials. Meanwhile, findings in a syngeneic mouse model of glioblastoma (GBM) indicated that antibody-targeted or certain lipid-complexed forms of DFM could create anti-tumor immunity by reprogramming tumor-associated microglia and macrophages (TAMs) from a tumor-promoting, Arginase 1 (Arg1)^high^, inducible nitric oxide synthase (iNOS)^low^ (M2-like) state into a tumoricidal, Arg1^low^, iNOS^high^ (M1-like) state [4,5]. The immunosuppressive M2-like polarization of TAMs is primarily promoted by the transcription factor Signal Transducer and Activator of Transcription 3 (STAT3) [6]. STAT3 also induces expression of immunosuppressive cytokines such as interleukin-10 (IL-10), IL-4, and IL-13 that reinforce the tumor-promoting environment. STAT3 also triggers the expression of the crucial enzyme Arg1 (a canonical marker of M2-like TAMs) [6,7,8]. In parallel, IL-10 inhibits a second transcription factor, STAT1, by suppressing its expression and phosphorylation [6,7,8].

Inhibition of STAT3 reverses this axis, allowing STAT1 induction and activation and downstream expression of iNOS (or NOS2), monocyte chemoattractant protein-1 (MCP-1), and IL-12, which are all hallmarks of tumoricidal M1-like TAMs [7,8,9]. Elevated nitric oxide (NO) production by iNOS is known to create a cytotoxic condition through the production of reactive nitrogen species [10], which would be expected to decimate the tumor from the inside. Prior research has also established that the TAM-released chemokine MCP-1 (also known as CCL2) crosses the blood–brain barrier (BBB) and binds to its receptor CCR2 on activated macrophages and natural killer (NK) cells in the peripheral system, thus promoting recruitment of activated, tumoricidal macrophages and NK cells into the GBM tumor [9,11,12]. Thus, targeted inhibition of STAT3 in microglia triggers a cascade of events that combine to eliminate GBM cells and GBM stem cells [9,12,13,14].

Activation of the M1 state or anticancer immunity has also been demonstrated pharmacologically in recent studies through selective and almost total inhibition of important proteins, such as Protein Tyrosine Phosphatase Nonreceptor type 22 (PTPN22), a key regulator of T-cell receptor (TCR) signaling [15]. While effective, such systemic activation of the immune system can lead to autoimmune side effects, which could be life-threatening for some individuals [16]. A selective immunomodulatory strategy that restricts macrophage reprogramming to the tumor site could achieve therapeutic efficacy while minimizing off-target inflammatory consequences. This underscores the importance of immune therapies that induce localized reprogramming of TAMs without triggering systemic immune dysregulation.

In the current study, we used a syngeneic, orthotopic mouse model of GBM and APG157DS, a drug under clinical investigation for multiple cancers. We report that APG157DS elicits long-term remission of GBM in 50% of the GBM-harboring mice. Furthermore, the presence of residual dead-cell clumps (scar tissue) on ex vivo MRI provided evidence that the GBM cells in the rescued mouse brains had been decimated by APG157DS. Additionally, we demonstrated that APG157DS treatment of GBM-harboring mice induced the expected M2 → M1 reprogramming of the TAMs and intra-tumor recruitment of activated NK and cytotoxic Tc cells as observed in our prior studies [5,7,17]. In contrast, APG157DS did not promote the formation of M1-like macrophages in cancer-free tissue, such as the spleen. Thus, we demonstrate that APG157DS is able to create anti-tumor immunity in GBM mice without precipitating autoimmune side effects. This study strongly supports earlier human clinical trials demonstrating the efficacy of APG157DS in head and neck cancer [18,19]. It also provides a mechanistic foundation for a pre-clinical study showing that APG157DS enhances the effect of anti-CTLA-4 in a mouse model of head and neck cancer [20].

## 2. Results

### 2.1. APG157DS Eliminates Mouse GBM GL261 Cells in Culture

**Figure 1:**
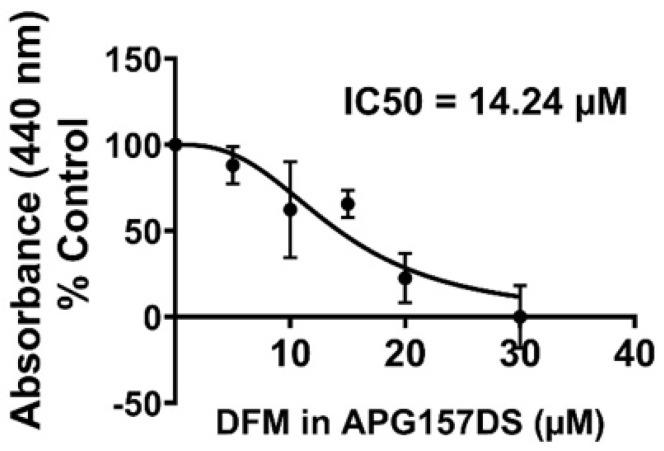
IC_50_ for DFM in APG157DS. GL261 cells in quadruplicate wells were treated with vehicle (Control) or increasing concentrations of APG157DS in serum-free RPMI with insulin-transferrin- selenium (ITS) supplement for 96 h, followed by WST-1 assay. The IC_50_ was calculated on the basis that APG157DS contains 50% DFM.

In our published studies, we observed that DFM displays an IC_50_ of ~15 μM in eliminating the GL261 cells in culture [21]. Based on the DFM content in the drug product APG157DS, the calculated IC_50_ of 14.24 μM is consistent with our earlier studies [4,7] (Figure 1).

### 2.2. Prolonged APG157DS Treatment Elicited Long-Term Remission of Mouse GBM

**Figure 2:**
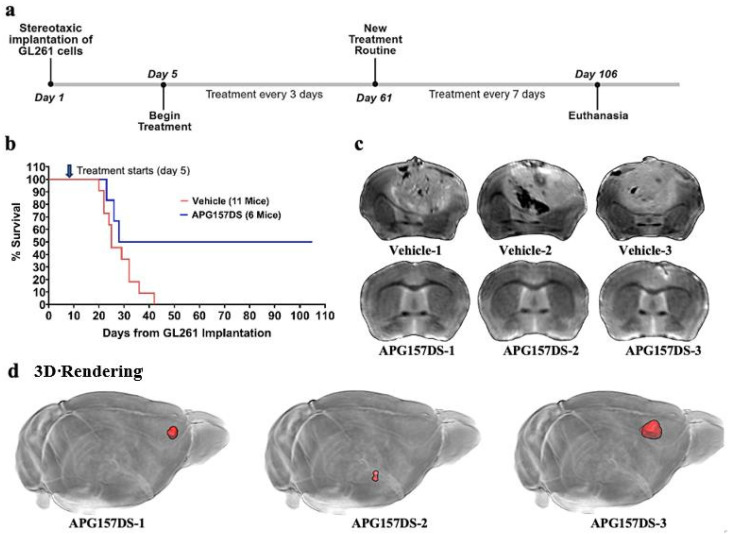
APG157DS treatment elicits long-term remission of glioblastoma (GBM) in a syngeneic mouse model of GBM. (**a**) Experimental plan. (**b**) Kaplan–Meier graph of mice intravenously treated with APG (4 mg in PBS per mouse per treatment). (**c**) MRI scans of mouse brains: Upper row: Vehicle-1, 2, 3: Brain tumors in vehicle-treated mice at euthanasia. APG157DS-1, 2, 3: Tumor-free brains of APG157DS-treated mice that attained long-term remission from GBM. (**d**) 3D rendering of the APG157DS-treated brains showing red clumps of scar tissue (dead tumor cells, pseudocolored red).

To test the efficacy of APG157DS in eliminating GBM, we implanted 20,000 GL261 cells per mouse orthotopically, in 3–5-month-old male mice on day 1. Subsequently on day 5, the mice were randomly divided into a “Vehicle Group” of eleven mice and an “APG157DS group” of six mice. The vehicle mice received 200 μL PBS intravenously every 72 h, whereas the APG157DS-treated mice received 4 mg of the drug substance in PBS intravenously every 72 h. From day 61, the frequency of dosing was decreased to once every seven days until day 106; at that point, the surviving mice were euthanized. While all eleven vehicle-treated mice progressed to humane endpoints by day 42, APG157DS treatment resulted in complete and sustained remission in three out of six mice, persisting through day 106 (Figure 2). Ex vivo MRI scanning of three vehicle brains showed large tumors, but similar scans of the three surviving mice treated with the test drug APG157DS revealed no tumors. 3D rendering of all three brains of mice treated with APG157DS revealed only scar tissue lumps (dead tumor cells). Glioblastoma is known for its infiltrative nature, with tumor cells often migrating beyond the original site of formation. Given the observed tumor-selective immune activation, it is plausible that repolarized TAMs continued to eliminate disseminated tumor cells at distant sites. Supporting this, 3D MRI analysis of the APG157DS-treated brains revealed two distinct regions of scar tissue located away from the primary GL261 implantation site (Figure 2d), consistent with immune-mediated clearance of migrating tumor cells.

### 2.3. APG157DS Treatment Stimulates Reprogramming of the TAMs from the M2-like to the M1-like State

In vitro analysis demonstrated that APG157DS exhibits an IC_50_ of 14.24 μM against GL261 glioma cells (Figure 1). However, previous pharmacokinetic analyses indicate that while peripherally delivered diferuloylmethane (DFM) does enter the brain, the concentration achieved is only about 30 nM [22]—a level far lower than the IC_50_ of DFM for the GL261 cells in vitro (~15–20 μM). Additionally, our mouse studies have also shown that the maximum plasma concentration of DFM transiently achieved in mice is 600 nM, which is far below its IC50 for GL261 cells [5]. Thus, the direct elimination of the GL261 tumor by DFM is unlikely. One alternative and more plausible mechanism to explain the observed effects at lower concentrations of DFM may involve APG157DS-mediated reprogramming of the TAMs within the GBM tumor from the tumor-promoting M2-like state to the tumoricidal M1-like state previously reported in our earlier studies [4,5].

**Figure 3:**
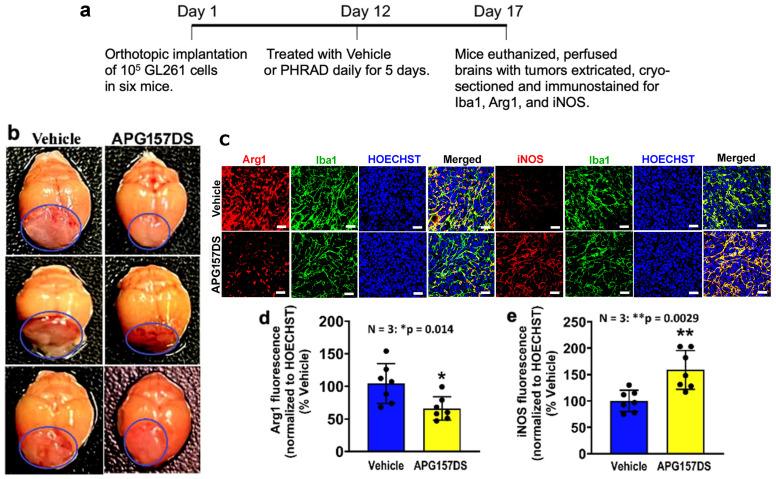
APG157DS treatment brings about reprogramming of tumor-associated microglia and macrophages (TAMs) from the tumor-promoting M2 to the tumoricidal M1 state. (**a**) Experimental plan. (**b**) Circled area in each panel shows the location of the hemorrhaging GBM tumor. The tumors were harvested, fixed, cryosectioned, and then the 30 µm sections were used for IHC analysis. (**c**) Confocal images of Iba1+ TAMs expressing high levels of either Arg1 (the M2-type) or iNOS (the M1-type). Cell nuclei were stained with HOECHST 33342. (**d**,**e**) Graphical presentation of the data with statistics is shown in (**c**). (**d**) Arg1 APG157DS vs. vehicle: * *p* = 0.0143 (three mice per treatment group, N = 3); (**e**) iNOS APG157DS vs. vehicle: ** *p* = 0.0029 (N = 3). Scale bar: 25 μm. Secondary antibody controls are presented in Figure S3 of the Supplementary Information.

To test the mechanism of APG157DS-mediated elimination of mouse GBM tumor in Figure 2, we conducted a shorter duration APG157DS treatment such that the GBM tumor was still expected to be present at the end of this five-day treatment. In this experiment, we also implanted a higher number of GL261 cells (10^5^ instead of 20,000) and started the vehicle or APG157DS treatment later, at day 12 (instead of day 5) when we expected the GBM tumor to be larger and more suitable for IHC analysis. None of the mice tested for reprogramming of TAMs, including those that were APG157DS-treated, were in remission and, therefore, all GL261-implanted mice harbored GBM tumors. The immunosuppressive cytokine IL-10 was induced in the vehicle-treated Iba1+ TAMs, but it was sharply inhibited in the APG157DS-treated TAMs (Figure S1). Consistent with this observation, APG157DS treatment also elicited a dramatic switch in the polarity of the Iba1+ TAMs in the GBM tumor from the tumor-promoting M2 to the tumoricidal M1 state as shown by an APG157DS-mediated decrease in Arg1 (marker for M2) and an increase in iNOS (marker for M1) staining (Figure 3).

### 2.4. APG157DS Treatment Induces Intratumoral Recruitment of Activated Natural Killer Cells and Cytotoxic Tc Cells

**Figure 4:**
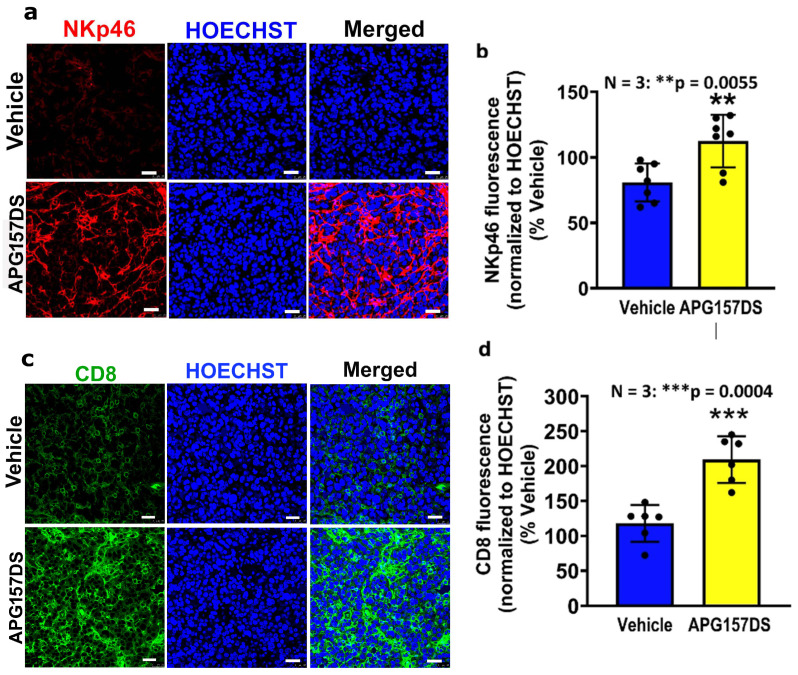
APG157DS treatment induces recruitment of NKp46+ natural killer (NK) cells and CD8+ Tc cells into the GBM tumor microenvironment. Parallel cryosections from Figure 3 were stained using antibodies against NKp46 (for activated NK cells) and CD8 (for activated Tc cells) to monitor intratumoral recruitment of NK and Tc cells. (**a**,**b**) The GBM sections from the APG157DS-treated mice show a dramatic increase in intratumoral recruitment of NK cells (** *p* = 0.0010, N = 3). (**c**,**d**) Similarly, the GBM sections from the APG157DS-treated mice show a sharp increase in recruited Tc cells (*** *p* = 0.004, N = 3). Scale bar: 25 μm.

In our earlier publications, we had observed that other DFM-based formulations induced both M2 → M1 reprogramming of TAMs and intratumoral recruitment of activated (NKp46+) natural killer (NK) cells and cytotoxic (CD8+) T (Tc) cells [5,7,23]. To determine whether the antitumor activity of APG157DS also followed a similar mechanism, we probed parallel tumor sections analyzed in Figure 3 with antibodies against NKp46 and CD8. We observed a sharp increase in both NKp46-positive NK cells and CD8+ Tc cells in the same GBM tumor sections obtained after the five-day APG157DS treatment of mice (Figure 4a–d).

### 2.5. APG157DS Treatment Elicits Significant Inhibition of Vascular Endothelial Growth Factor and Hypoxia-Inducible Factor 1 Alpha in the Tumor

Most growing solid tumors, including GBM, develop hypoxic cores as they grow, which triggers angiogenesis through the induction of secreted factors such as vascular endothelial growth factor (VEGF) and hypoxia-inducible factor 1 alpha (HIF-1α) in the tumor cells [24,25]. Although the M1-like TAMs and the recruited NK and Tc cells are expected to eliminate the GBM tumor cells, we sought to determine if the antitumor activity of APG157DS also involved suppression of expression of VEGF and HIF-1α. Although not as pronounced as the repolarization of TAMs and the recruitment of NK and Tc cells, we observed a significant inhibition of both VEGF and HIF-1α in parallel sections obtained from the APG157DS-treated mice as presented in Figure S2a–d. It is generally understood that HIF-1α moves from the cytoplasm to the nucleus to function as a transcription factor, but in some situations, it is present in the cytoplasm [26]. Also, Wu and coworkers have shown that HIF-1α is expressed in both the cytoplasm and the nucleus in gallbladder cancer cells and they found that the presence of both cytoplasmic and nuclear HIF-1α were correlated to microvessel density (indicating angiogenesis) in gallbladder cancer cells [27]. Our results indicate an abundant presence of HIF-1α in both the cytoplasm and nuclei of the GBM cells in the vehicle-treated mice, which was moderately suppressed in the APG157DS-treated mice. These results suggest that APG157DS may exert complementary antitumor effects by attenuating tumor angiogenesis.

### 2.6. APG157DS Treatment Does Not Precipitate Systemic Autoimmune Effect: M1 Macrophages Are Suppressed in the Spleen of Cancer-Free Mice

**Figure 5:**
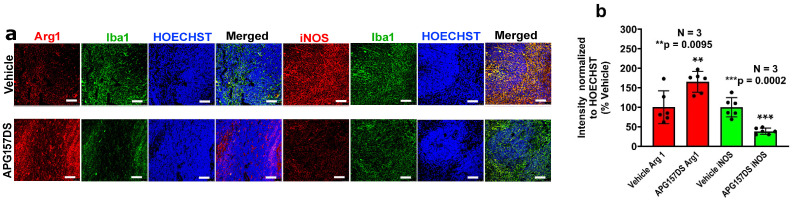
APG157DS treatment suppresses M1 macrophages in cancer-free spleen. Healthy, cancer-free mice were treated once with APG157DS (4 mg) in PBS (i.p.) and 24 h later, the mice were perfused under deep anesthesia with PBS and then 4% paraformaldehyde. (**a**) Cryosections of the spleens were subjected to IHC to probe for Arg1 and iNOS in Iba1+ macrophages. (**b**) Vehicle Arg1 vs. APG157DS Arg1: ** *p* = 0.0095; Vehicle iNOS vs. APG157DS iNOS: *** *p* = 0.0002. (N = 3 mice per treatment). Scale bar: 25 μm.

It was discussed in the introduction that some recent studies have reported that a pharmacological inhibitor of Protein Tyrosine Phosphatase Nonreceptor type 22 (PTPN22), a key regulator of T-cell receptor (TCR) signaling, can also induce M1 polarization of macrophages [15]. Unfortunately, such a systemic inflammatory condition throughout the body is likely to lead to autoimmune side effects, which could be life-threatening [16]. Therefore, repolarization of macrophages and microglia into the M1 state would be safe only if it occurred selectively within the tumor and not in the cancer-free tissue in the rest of the body. To evaluate the selectivity of APG157DS-induced immune modulation, we subjected cancer-free mice to APG157DS treatment as shown in Figure 5 and examined the polarity of the macrophages in the spleen. After 24 h, APG157DS (4 mg) treatment caused a significant inhibition of M1-type macrophages in the spleen (Figure 5). Therefore, APG157DS elicits antitumor immunity by turning the TAMs against the GBM tumor while suppressing such inflammatory M1-type macrophages in the normal peripheral tissue.

## 3. Discussion

Glioblastoma (GBM) remains among the most challenging solid tumors to treat, with limited success from conventional cytotoxic therapies or immune checkpoint inhibitors, largely due to its immune-privileged microenvironment, low neoantigen burden, and profound myeloid-driven immunosuppression [28]. Traditional approaches such as chemotherapy or T-cell-directed immune checkpoint inhibitors (e.g., PD-1 blockade with nivolumab or pembrolizumab) have demonstrated minimal benefit in GBM [28]. Advanced strategies such as CAR T-cell therapy, while transformative in hematologic malignancies and select peripheral tumors, have not translated into durable responses in GBM, owing in part to poor T-cell infiltration, antigen heterogeneity, and an immunosuppressive tumor microenvironment [28,29,30,31].

Despite such observations, mechanisms that dampen T-cell–mediated activity—such as immune checkpoint pathways and the involvement of CD4+ cells—are often considered the main drivers of immunosuppression in GBM. Based on the studies presented in the preceding section, immunosuppression driven by the overwhelming number of myeloid-derived cells such as microglia and macrophages in the GBM tumor holds the key to the immunosuppressive TME [28]. Thus, the M2-type TAMs formed by the cytokines released by the GBM cells are at the center of the immunosuppressive TME. Since DFM is central to the APG157DS-mediated reprogramming of TAMs, the current study rests on the data from our prior publications using various versions of DFM [4,5,7]. These articles show that DFM treatment of GBM-harboring mice elicits marked inhibition of STAT3 and its downstream immunosuppressive cytokine IL-10 and a simultaneous induction of STAT1 and its downstream M1-linked cytokine IL-12 [7]. It is also established through multiple studies by various teams that the downstream effector molecule in the STAT3-IL-10 axis is Arg1, the M2 marker, and the downstream effector in the STAT1-IL-12 axis is iNOS, the M1 marker [6,7,8,9,12,13,14]. Since our earlier studies have already used IHC and flow cytometry (FC) to confirm that DFM elicits inhibition of IL-10 and induction of IL-12 in the TAMs, the current study has focused on the corresponding downstream signaling molecules Arg1 and iNOS, respectively. Nonetheless, we have also confirmed that APG157DS causes inhibition of IL-10 in the Iba1+ TAMs in the GBM-harboring mice (Figure S1). The major objectives of this study were to (1) show that APG157DS was highly potent in decimating GBM and (2) explore the likely mechanism of this protective activity, involving the M2 → M1 reprogramming of the GBM TAMs.

We have observed a dramatic protective effect of APG157DS in an orthotopic, syngeneic model of GBM. Based on our earlier published studies on various forms of DFM and DFM derivatives, we were confident we would observe a strong protective effect of APG157DS. This prompted us to preform rigorous ex vivo MRI analysis of the brains of a sampling of GBM brains from the morbid mice and the brains of all mice that were rescued by APG157DS treatment (Figure 2). Given a limit on how many brains could be subjected to ex vivo MRI, we decided to include only six APG157DS-treated mice in the long-term treatment. A sampling of three brains from all eleven vehicle-treated mice that reached the humane endpoint were subjected to MRI to clearly visualize the extent of the GBM tumor in the succumbing brains. In addition, the brains of all three APG157DS-rescued mice out of six were scanned by MRI to confirm that the GBM tumor cells had indeed been decimated. Thus, the number “six” was important because each mouse represented 16.7 percent of the entire group. Therefore, although this number was not derived from a power analysis, decimation of GBM in three of six APG157DS-treated mice amounted to an impressive rate of 50% long-term rescue in this group. In sharp contrast, the termination of all eleven mice in the “Vehicle” cohort further confirmed that the observed 50% rescue in the APG157DS group was not due to spontaneous regression of GBM. Furthermore, the rigorous 3D-rendered MRI images (Figure 2) confirmed that GBM cells in these rescued brains had indeed been eliminated by APG157DS, leaving behind some dead cells in scar tissue clumps.

In this context, we investigated the immunomodulatory effects of APG157DS, a novel multi-component oral agent that includes diferuloylmethane (DFM) and is under clinical evaluation for several cancers [2,18,19]. Our findings demonstrate that APG157DS elicits robust innate immune reprogramming, intratumoral recruitment of cytotoxic immune cells, and durable tumor remission in a subset of treated animals. These results support the continued development of APG157DS as a selective immunomodulator for immune-refractory tumors such as GBM.

DFM, a key active ingredient in APG157DS, has an in vitro IC_50_ of 14.24 μM against GL261 cells (Figure 1), but its systemic administration results in brain concentrations of only ~30 nM [22]. This >500-fold discrepancy between cytotoxic threshold and achievable tissue exposure strongly argues against a direct anti-proliferative mechanism and instead supports an immune-mediated effect. Consistent with this argument, human clinical studies have shown that DFM from transoral APG157DS reaches a mean serum concentration of 13 nM at 3 h, which is far below the IC_50_ of DFM, yet tumor elimination is observed in such studies [18]. Thus, such observations are consistent with our prior findings and those of others showing that DFM can reprogram macrophages in vivo at sub-cytotoxic concentrations [4,5,12]. Future studies will integrate systemic and intratumoral drug concentration measurements with time-matched immune profiling (e.g., STAT3/STAT1 regulation, macrophage polarization markers) to quantitatively link APG157DS exposure with immune modulation.

Consistent with our prior studies, we have observed a marked phenotypic shift in tumor-associated macrophages (TAMs) from Arg1^high^, iNOS^low^ (M2-like) to Arg1^low^, iNOS^high^ (M1-like) following APG157DS treatment [5]. Prior studies indicate that M2 polarization is driven by STAT3 and reversed by its inhibition, which relieves suppression of STAT1, IL-12, and iNOS [6,7,8,9,13]. In line with this information, our first publication in a series showed that GBM cells from both from human and mice express CD68 at levels far higher than those of microglia and macrophages. By targeting DFM using a CD68 antibody to mouse GBM, we could both induce and activate the M1-linked STAT1 and also rescue 50% of the GBM mice [4]. Our subsequent study confirmed that a lipid-complexed form of DFM efficiently inhibited the M2-linked, immunosuppressive transcription factor STAT3 [7]. Since APG157DS is an orally administered form of the same DFM and its downstream effect on the STAT3–IL-10–STAT1–IL-12–Arg1–iNOS axis has been shown here as inhibition of Arg1 (M2 marker) and induction of iNOS (M1 marker) in the Iba1+ TAMs (Figure 3), our future studies will mainly focus on the role of the dichotomous cytokine IL-6 which may play a central role in eliciting M2 programming of TAMs by inhibiting M1 programming of macrophages in the noncancerous peripheral organs (explained later).

Intratumoral recruitment of NKp46+ natural killer cells and CD8+ cytotoxic T cells has been observed both by us as well as others [7,23,32,33,34,35,36]. However, the effector function of these cells was not assessed in this study. It remains unknown whether recruited CD8+ T cells and NK cells exhibit cytotoxic activity, cytokine production, or memory potential. Since the adaptive immune system has been the focus of most anticancer strategies, the fact that the innate immune macrophages and microglia are more numerous than T cells inside a typical GBM has been largely ignored. Whereas our prior studies have established that the recruited NK cells play an important role in DFM-evoked reprogramming of the TAMs [7], the role of far fewer recruited Tc cells or the immunosuppressive Treg cells may have an indirect effect on the innate immune cells, which will be analyzed in our future studies. As mentioned earlier, T cell activation strategies have not been effective against GBM in many studies so far. This justifies our current focus on revealing the role of the innate immune system, which plays an important role in tumor immunity.

The GL261 orthotopic syngeneic model is widely used for preclinical immunotherapy studies due to its immunocompetence and reproducibility [37,38]. It lacks the isocitrate dehydrogenase 1 (IDH1) mutation found in many human gliomas; however, this may not be critical for innate immune-targeting agents like APG157DS. Supporting this, prior studies using IDH1-mutant GL261 variants showed prolonged survival following innate immune stimulation via STING agonists, effects abrogated by NK cell depletion [39,40]. Thus, our data that APG157DS induces microglia/macrophage repolarization and innate effector recruitment provide further support for this approach. Nevertheless, GL261 tumors harbor a KRAS mutation, which is absent in most human GBMs [41]. Therefore, additional models—such as a syngeneic model created using the KRAS-wild-type mouse GBM cell line CT2a or genetically engineered IDH1-mutant gliomas—would be helpful in confirming these findings in more clinically relevant settings.

A key advantage of APG157DS is its spatially selective immune modulation. Whereas systemic immune activators like a PTPN22 inhibitor are likely to induce widespread M1 polarization and inflammation [15], APG157DS only elicited suppression of M1-type macrophage reprogramming in the spleen of healthy mice. This finding aligns with previous reports suggesting a restricted effect of DFM analogues within the tumor microenvironment [4,7,11,12]. However, this selective activity was assessed at a single time point in one peripheral organ. A rigorous preclinical safety assessment would require longitudinal monitoring of inflammatory markers and histopathology across multiple organs (e.g., liver, lung, gut, skin), as well as evaluation of autoimmune markers.

In addition to innate immune remodeling, we observed reduced VEGF and HIF-1α expression in tumors treated with APG157DS, consistent with antiangiogenic activity [24,25]. Tumor hypoxia and neovascularization contribute to GBM progression and immune evasion, suggesting that vascular normalization may enhance immune infiltration and therapy responsiveness. The dual immunomodulatory and antiangiogenic properties of APG157DS may offer synergistic therapeutic potential, particularly in combination with agents like bevacizumab.

The dichotomous role of APG157DS in the tumor versus in the noncancerous spleen raises mechanistic questions. The anti-inflammatory role of DFM has been established in many studies [42,43,44,45], but a few recent reports on the pleiotropic cytokine IL-6 shed light on the likely mechanism that explains this dichotomy [46,47,48]. Wang and coworkers have demonstrated that endothelium-released IL-6 cooperates with colony-stimulating factor-1 (CSF-1) to trigger alternative activation of GBM TAMs (i.e., M2) through HIF-2α [46]. It is thus reported that IL-6 functions through a membrane-bound receptor to induce immunosuppression by inducing M2 TAMs in the tumor, whereas it signals through a soluble receptor to trigger inflammation in noncancerous tissue in the peripheral organs [46,47,48]. DFM is known to inhibit IL-6 signaling in both pathways [49,50,51]. This might explain why APG157DS could play opposing roles in GBM TAMs and the macrophages in the spleen.

## 4. Materials and Methods

### 4.1. Animals

The experiments involved adult male C57BL/6 mice, aged 3 to 5 months. These mice were bred at the College of Staten Island (CSI) Animal Care Facility and housed under a controlled 12-h light/dark cycle, with free access to both food and water. All animal-related procedures conducted in this study adhered to the ethical guidelines set forth by the Institutional Animal Care Committee (IACUC) at the College of Staten Island.

### 4.2. Cell Culture

GL261 mouse glioblastoma cells were maintained in RPMI 1640 (RPMI) medium supplemented with 10% fetal bovine serum (FBS) and 1% gentamicin. Prior to and during drug exposure, the cells were maintained in serum-free RPMI medium supplemented with 1% insulin-transferrin-selenium (ITS) and 1% gentamicin [4].

### 4.3. Implantation of Cancer Cells in Mice

GL261 mouse glioblastoma cells (2 × 10^4^ or 10^5^) were implanted stereotactically in the right forebrain of C57BL/6 mice, following our earlier reports [4,5,7,21]. The GL261-implanted mouse model has been thoroughly characterized by magnetic resonance imaging (MRI) and numerous biological studies [5,7,21,38,41,52].

### 4.4. Determination of IC_50_ Using WST-1 Assay

Cellular proliferation was assessed using the WST-1 assay. Briefly, 3000 cells were seeded per well in a 96-well plate and incubated overnight at 37 °C in a humidified incubator with 5% CO_2_. As previously described, stock solutions of APG157DS drug substance (containing 50% DFM) were prepared in dimethyl sulfoxide (DMSO), then serially diluted in RPMI medium supplemented with 1× insulin-transferrin-selenium (ITS) (Thermo Fisher Scientific, Springfield, NJ, USA). The final DMSO concentration was ≤0.1%. RPMI-ITS of the same DMSO content was used to treat cells in the control wells. Cells in quadruplicate wells were treated with either RPMI-ITS medium or increasing concentrations of APG157DS in RPMI-ITS medium and then incubated for 96 h in a humidified, 5% CO_2_ incubator. Following this treatment, the medium in each well was removed, and the cells were gently rinsed three times with phosphate-buffered saline (PBS). Next, the cells were incubated at 37 °C for 45 min with a 10% (*v*/*v*) solution of WST-1 (Clontech, Mountain View, CA, USA) diluted in serum-free RPMI as detailed in our prior publication [6]. Optical density was measured at 440 nm using a microplate reader. All experiments were carried out three times to determine the IC_50_ of the drugs for GL261 cells. GraphPad Prism (version 11.0.2) was used for graphical analysis [4,5,7,23,53].

### 4.5. Treatment of Animals

**Long-term survival:** APG157DS contains 50% DFM [18,19,20,54]. To test the ability of APG157DS to eliminate GBM, seventeen mice were implanted with 2 × 10^4^ mouse GBM GL261 cells orthotopically on day 1. On day 5, six randomly selected mice were used for APG157DS treatment and eleven mice were used for vehicle treatment. Similar to our earlier studies, vehicle (200 μL of PBS) or APG157DS (4 mg, 5.4 μmol of DFM, finely dispersed by vigorous vortexing in 200 μL of PBS) was administered intravenously every 72 h starting from day 5 as shown in Figure 2a. For any mouse, the humane endpoint was reached when a mouse either lost 15% of body weight and/or was severely hunched, stopped eating or moving. At that point, the mouse was placed under deep anesthesia and, for MRI scanning, perfused through the heart with PBS first and then 4% paraformaldehyde followed by decapitation. The experimenters were not blinded to the vehicle and APG157DS groups, but they were blinded to the identity of each individual mouse within a treatment cohort.

As for the dose of APG157DS, in our earlier studies, we infused (i.p.) 2 mg of a phytosomal preparation of DFM containing ~10% DFM (~0.2 mg) to rescue GBM-harboring mice [7]. Since APG157DS contained only the extracted components from a natural substance, we did not expect the presence of a large amount of phytosomal-type lipids to protect DFM. Therefore, we used a larger dose of 4 mg of APG157DS, which would introduce about 2 mg of DFM (50% in APG157DS) per mouse per treatment.

### 4.6. Ex Vivo MRI

At the experimental endpoints (humane or at day 106 for the surviving animals), mice harboring GBM tumors were placed under deep anesthesia using a cocktail of Ketamine (100 mg/kg) and Xylazine (10 mg/kg). They were then perfused through the heart with PBS followed by 4% paraformaldehyde in PBS. After decapitation with a guillotine, the heads were stored in 4% paraformaldehyde in PBS at 4 °C until similar heads from other cohorts were obtained. Three out of six APG157DS-treated mice appeared symptom-free at day 106, when they were similarly anesthetized, perfused through the heart, decapitated, and their heads stored in 4% paraformaldehyde at 4 °C until ex vivo magnetic resonance imaging (MRI). Similar to previous studies [55,56], we employed a high-throughput ex vivo MRI approach, enabling simultaneous scanning of multiple samples using extended overnight imaging time. Each whole head was de-skinned, with ears and facial muscles trimmed off, including the removal of the mandible to fit and secured each of the four heads with glue (Krazy Glue; Elmer’s Products Inc., Columbus, OH, USA) within the four sides of the plunger of an 80 mL syringe. The plunger served as a four-compartment divider, with each head secured in one quadrant.

Polyethylene tubing filled with copper-doped water was used to identify the orientation and location of the samples during the MRI scan. A single tubing was placed next the first sample, while the fourth sample was identified with a double-tubing placed in the neighboring quadrant counterclockwise. The plunger was then inserted into the syringe and immersed in Fomblin (Ausimont, Thorofare, NJ, USA), a proton-free perfluoropolyether fluid. Fomblin matches the magnetic susceptibility of biological tissues without producing background signals, significantly improving shimming. Specifically, the dark background created by Fomblin allows the shimming process to focus entirely on correcting the homogeneity of the sample itself, free from interference by the surrounding background. Additionally, susceptibility matching between the tissue and Fomblin reduces magnetic field distortions that typically arise from air-tissue interfaces, further minimizing artifacts. This dual effect ensures clearer and more accurate MRI images while preventing dehydration.

The sealed syringe with its tip oriented towards the top, was placed in a vacuum chamber to degas the sample for at least two hours, preventing bubbles or air pockets within the skull that could compromise image quality. MRI scans were performed on a Biospec 70/30 micro-MRI system (Bruker, Billerica, MA, USA) equipped with a zero-helium boil-off 300 mm horizontal bore 7-Tesla (7-T) superconducting magnet (300 MHz) based on ultra-shield refrigerated magnet technology (USR). The magnet was interfaced to an actively shielded gradient coil insert (Bruker BGA-12S-HP; OD = 198 mm, ID = 114 mm, 660 mT/m gradient strength, 130-μs rise time) and powered by a set of high-performance gradient amplifiers (IECO, Helsinki, Finland) operating at 300 A/500 V. This setup was controlled by an Avance-3HD console operated under Paravision 7.0 and TopSpin 3.1.

The MRI setup utilized a Bruker circularly polarized (CP) radiofrequency (RF) birdcage resonator, typically used for scanning a mouse body (inner diameter = 40 mm), which conveniently closely fits the diameter of an 80 mL syringe. A three-dimensional (3D) *T*_2_-weighted Fast-Spin Echo (FSE) pulse sequence, known for its excellent gray/white matter tissue contrast was used to help delineate the tumor volume via the expected tissue edema, hemorrhage, and necrosis typically associated with brain tumors with the latter at an advanced stage. Simultaneous scanning of the four whole heads generated datasets with 150 µm isotropic resolution where the effective echo time (TEeff) was 60 ms, the echo spacing (ES) was 20 ms, and the repetition time (TR) was 1200 ms. The acceleration factor (AF) was 8, with the number of averages (Nav) being 2. The matrix size (Mx) was 256 × 256 × 256 with antialiasing along the slice phase encoding direction (aa3) of 1.36, resulting in aa3 Mx of 256 × 256 × 348. The field-of-view (FOV) was 38.4 mm × 38.4 mm × 38.4 mm, and the bandwidth (BW) was 20 kHz (78.13 Hz/pixel), resulting in an overall imaging time of 7 h and 27 min.

Following the completion of the acquisition, all datasets were processed and converted into NIfTI file format to facilitate seamless portability, visualization, and post-analysis using various open software such as NIH ImageJ2 (version 2.14.0/1.54g) or equivalents like Fiji (version 6). The 150 µm isotropic resolution facilitated 3D image registration and eased analysis of the tumor volume quantification and rendering using the commercial software Amira (version 6.7) (Bordeaux, France). The difference in tissue contrast between the tumor and the surrounding brain tissue enabled precise segmentation and isolation of the tumor mass within the whole brain. This image analysis also enabled 3D segmented rendering of the tumor within the whole head anatomical context.

**Hematoxylin-Eosin (H&E) staining subsequent to ex vivo MRI:** Following MRI scanning of a sampling of brains from three “Vehicle” mice and all three of the APG157DS-rescued GBM mice were subjected to H&E staining according to our published protocols [53]. The position and boundaries of the GBM in the “Vehicle” mice were clearly visible in the stained images (Figure S4).

**Short-term treatment to study reprogramming of TAMs:** Each mouse in the short-term-survival group was implanted with 10^5^ GL261 cells on day 1. Three GL261-implanted mice were used in each of the vehicle and APG157DS groups. For this 5-day treatment, each APG157DS injection (i.v.) contained 4 mg of APG157DS drug substance finely dispersed in PBS. The vehicle mice received 200 μL of PBS (i.v.). Daily treatment commenced on day 12, and over the course of 5 days, the mice received intravenous (i.v.) treatments of 200 μL of PBS or APG157DS in PBS per mouse every 24 h. After five doses (day 16) of the short-term treatment, the mice also received intranasal delivery of DyLight-CD68 Ab for near-IR scanning, as previously reported [4,21]. Subsequently, on the sixth day (day 17), mice from all three groups were sacrificed, and their brains were near-IR-scanned using an Odyssey scanner (Li-COR, Lincoln, NE, USA) as a second mode of identification of GBM tumors [4,21,23]. Next, the hemorrhaging tumors were visually identified and resected from the brain, fixed in 4% paraformaldehyde, cryosectioned, and processed for IHC as described in our earlier reports [4,5,7]. It should be noted that in vivo scanning by bioluminescence or ex vivo scanning by magnetic resonance imaging (MRI) cannot directly guide tumor resection. Only visual inspection of the hemorrhagic tumor area allows resection of the tumor from the remaining brain tissue.

### 4.7. Antibodies, Dilutions and Immunohistochemistry (IHC)

Iba1 (goat IgG) (C20) (sc28530) (1:50), NKp46 (rabbit IgG) (sc-292796) (1:100), anti-STAT1 (rabbit IgG) (sc-592) (1:100), anti-P-Tyr^701^-STAT1 (mouse IgG) (sc-8394) (1:100), anti-CD8-α (D-9) (mouse IgG) (sc-7970) (1:100), VEGF (P-20) Goat polyclonal IgG (sc-1836) (1:20), HIF-1α (H1a 67) mouse monoclonal IgG (sc-53546) (1:50) (Santa Cruz Biotechnology, Dallas, TX, USA). All antibodies were diluted in 2% goat serum and 2% rabbit serum and 0.1% Triton X-100 in PBS (GRT-PBS). Alexa Fluor 488 rabbit anti-goat, Alexa Fluor 568 goat anti-mouse, and Alexa Fluor 488 goat anti-mouse) (Invitrogen/ Thermo Fisher, Branchburg, NJ, USA) (1:1000 dilutions in GRT-PBS). For IHC, three mice per treatment were used to collect the tumor from each brain, fixed in 4% paraformaldehyde, and then processed following procedures detailed in our earlier publications [4,5,7]. Confocal microscopy was conducted using an SP8 Leica microscope. We conducted each drug treatment using three mice. From each mouse, three tumor sections were used for IHC. In each immunostained section, 2–3 randomly chosen tumor areas were imaged using confocal microscopy. The immunostained intensity of an antigen (e.g., NKp46) in each area was divided by the intensity of Hoechst staining (Sigma, St. Louis, MO, USA) of all nuclei in the same area and then each quotient was divided by the same values obtained from the control brain sections and expressed as % vehicle. In this way, the mean ± S.D. intensity for each antigen was obtained from a large number of confocal images. The 2° Ab controls showed no nonspecific staining (Figure S3).

## 5. Conclusions

This study provides mechanistic support for APG157DS as a selective immunomodulatory agent capable of reprogramming tumor-associated macrophages, recruiting cytotoxic effector cells, and inducing long-term tumor remission without triggering systemic immune activation. These effects appear independent of tumor mutation status, and are aligned with preliminary clinical findings in head and neck cancer patients [18,19,20,54]. However, to support regulatory development in GBM, future studies will address some outstanding questions. We will study the efficacy of APG157DS in other clinically relevant tumor models, such as those harboring IDH1 mutations and KRAS wild-type gliomas, e.g., CT2a GBM cells. We will also validate immune signaling pathways such as the STAT3/STAT1 axis and cytokine networks and quantify immune responses through T cells and NK cells. Finally, we will establish safety across multiple organ systems using chronic APG157DS treatments.

## Data Availability

The original contributions presented in this study are included in the article/Supplementary Material. Further inquiries can be directed to the corresponding author.

## Supplementary Materials

The following supporting information can be downloaded at: https://www.mdpi.com/article/10.3390/ijms27156687/s1.

## Abbreviations

APG-157DS (APG157DS); HOECHST (HOECHST33342); Arginase1 (Arg1); inducible nitric oxide synthase (iNOS); Vascular endothelial growth factor (VEGF); Hypoxia-inducible factor 1-alpha (HIF-1α); Magnetic resonance imaging (MRI); Glioblastoma (GBM); Signal Transducer and Activator of Transcription 3 (STAT3); Signal Transducer and Activator of Transcription 31 (STAT1); Roswell Park Memorial Institute (RPMI); Insulin-transferrin-selenium (ITS); Immunohistochemistry (IHC).
