# Supplementary material for: First-in-Class Immuno-Oncology Drug APG157DS Repolarizes Innate Immune Cells and Induces Durable Remission in a Syngeneic Glioblastoma Model
Source: Int J Mol Sci. 2026 Jul 27;27(15):6687. doi: 10.3390/ijms27156687 (PMC13466085; doi:10.3390/ijms27156687)
Supplement: Supplementary file 1 [file ijms-27-06687-s001.zip › ijms-4360176-supplementary.pdf]

Supplementary Information

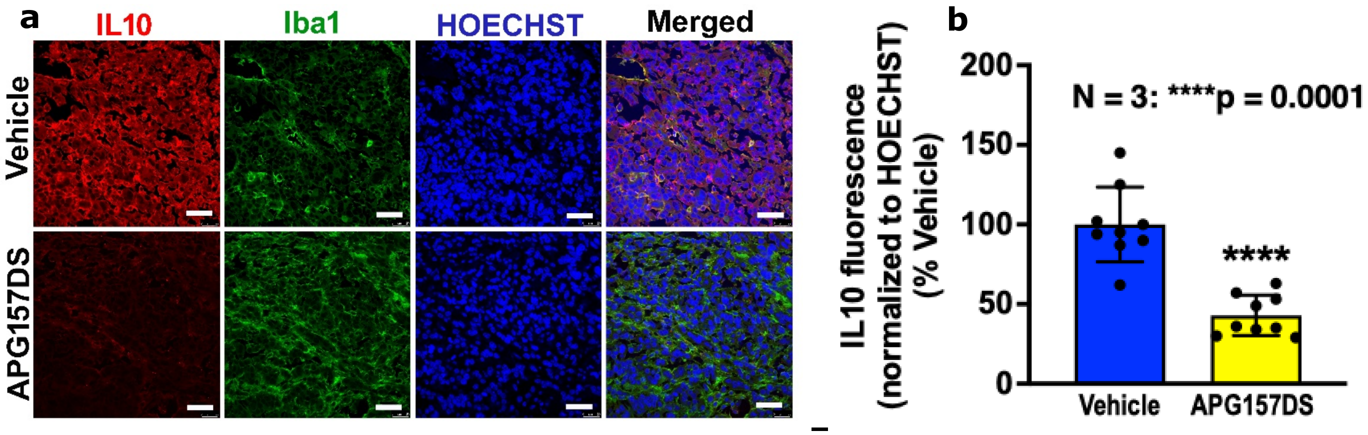

Figure S1. APG157DS treatment of GBM mice elicits inhibition of the immunosuppressive cytokine IL-10 in the Iba1+ TAMs. Scale bar: 25 µm.

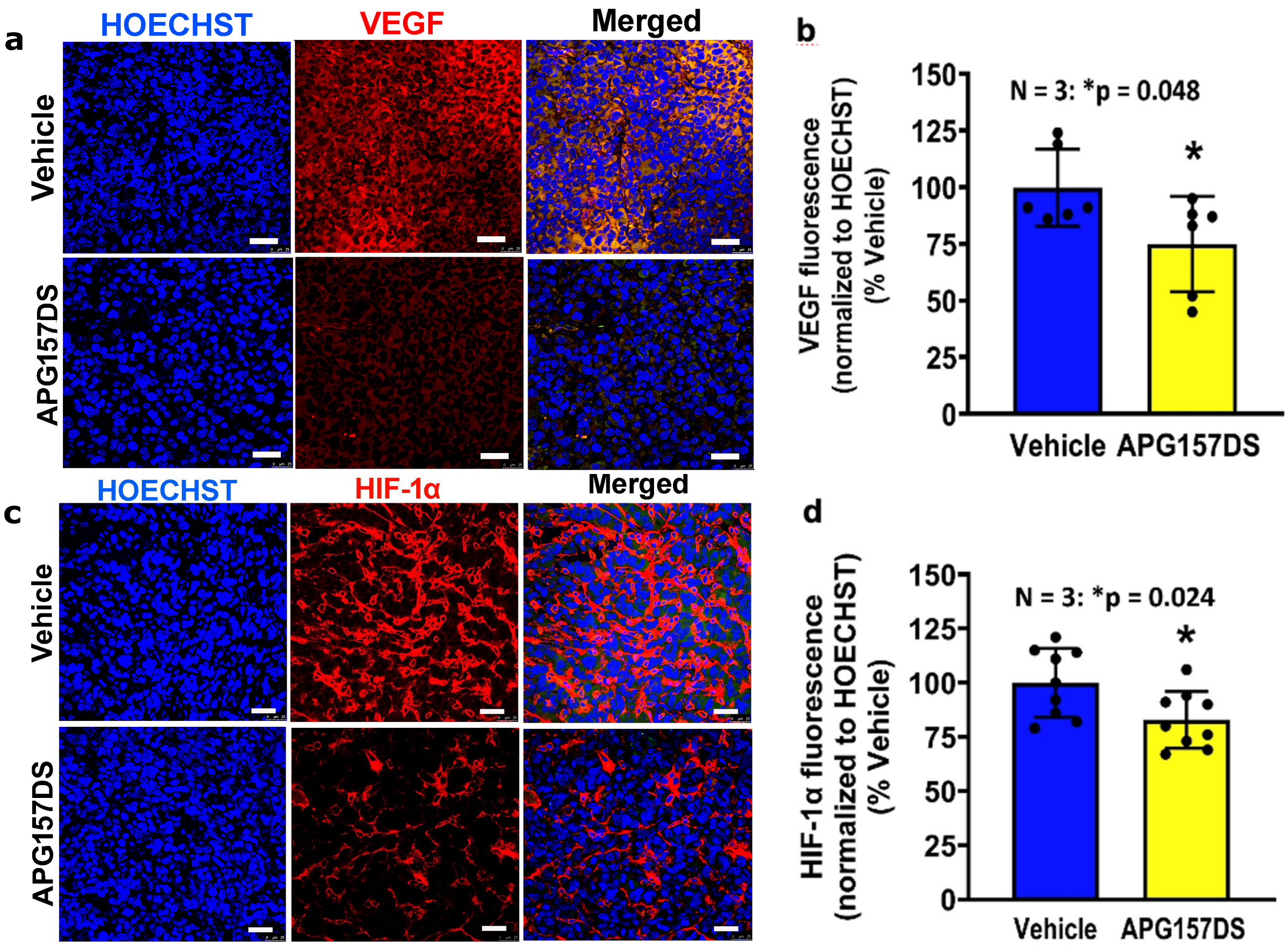

Figure S2. APG157DS treatment causes inhibition of VEGF and HIF-1α in the GBM tumor. Parallel cryosections from tumors shown in Figure 3 were probed for VEGF and HIF-1**α**. **(a)** and **(b):** Parallel tumor sections collected in Figure 3 show a significant inhibition of VEGF expression in the APG157DS-treated mice (* p = 0.048, N = 3). **(c)** and **(d):** Similarly, a significant inhibition of HIF-1**α** expression was observed in the tumor sections from the APG157DS-treated mice (* p = 0.024, N = 3). Scale bar: 25 µm.

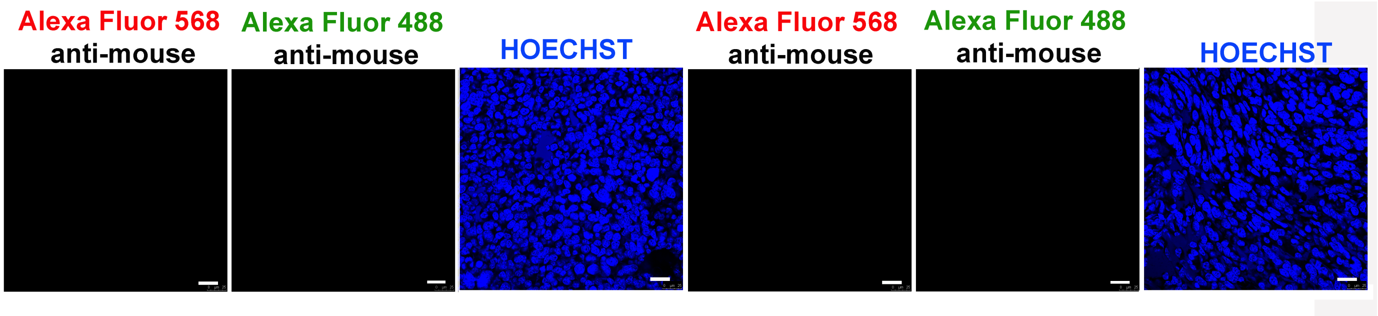

Figure S3. 2° Ab controls for Figure 5a. The same secondary antibodies were used for IHC data presented in other figures. Scale bar: 25 µm.

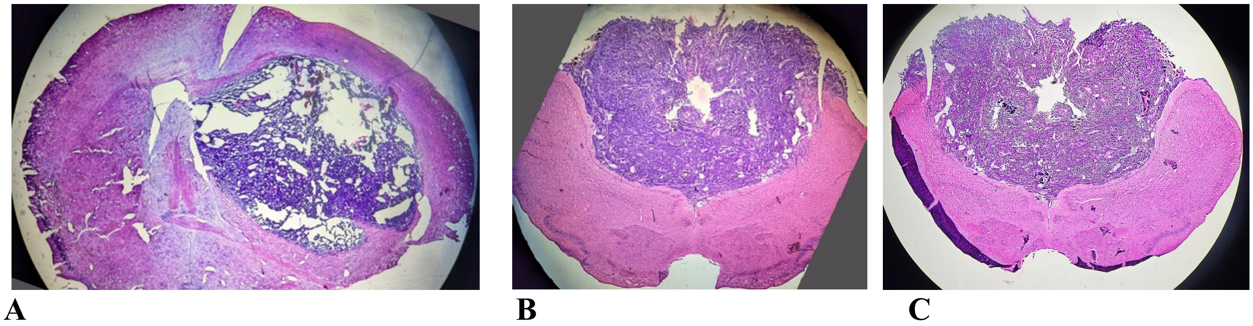

Figure S4. Hematoxylin-Eosin (H&E) staining of whole brain sections, showing hematoxylin-stained GBM cells surrounded by noncancerous tissue. In confocal images shown in Fig. 3-5, S1-3, the GBM region displays densely Hoechst-stained nuclei and is surrounded by less dense noncancerous cells. A-C show microscopic images acquired in brightfield at 2.5× magnification of H&E-stained GBM brain sections from three vehicle-treated mice at humane endpoints. All IHC images shown in Figures 3, 4, 5, S1, S2, and S3 were acquired at higher magnifications from such tumor regions.
